# Depalmitoylase ABHD16A negatively regulates the anti-hepatitis B virus activity of IFITM1

**DOI:** 10.1128/spectrum.03095-24

**Published:** 2025-05-28

**Authors:** Xin Wen, Mingyang Liu, Yali Fan, Junfei Xu, Zhaoyan Wang, Lin Mao, Weizhen Gu, Xuemeng Shi, Jun Xu

**Affiliations:** 1 College of Life Science, Zhengdong New District Longzi Lake Campus, Henan Agricultural University 70573, Zhengzhou, Henan, China; Xinxiang Medical University, Xinxiang, Henan, China

**Keywords:** HBV replication, IFITM1, ABHD16A, post-translational modification, palmitoylation

## Abstract

Interferon-inducible transmembrane (IFITM) proteins have been widely reported as antiviral factors against various viral pathogens. However, the mechanisms of IFITM regulation on hepatitis B virus (HBV), which induces chronic infection resulting in cirrhosis as well as liver cancer, remain poorly characterized. In the present study, we identified that HBV infection significantly increased the expression of IFITM1. To dissect the role of IFITM1 in HBV infection, we overexpressed IFITM1 in HepG2.215 cells and revealed that the replication of HBV was restricted by IFITM1. Recently, we demonstrated that the anti-RNA virus activity of IFITM proteins depends on palmitoylation modification, and the depalmitoylase α/β-hydrolase domain-containing 16A (ABHD16A) negatively regulates IFITM1 against RNA virus infection in human and porcine cells. However, whether ABHD16A-IFITM1 regulates DNA virus infection has not been researched. Here, by using co-immunoprecipitation (Co-IP) and acyl-PEGyl exchange gel-shift assay, ABHD16A-catalyzed depalmitoylation of IFITM1 was verified in HepG2.215 cells. In addition, we respectively knocked out IFITM1 and ABHD16A via CRISPR/Cas9 and showed that the anti-HBV activity of IFITM1 was negatively regulated by ABHD16A through depalmitoylation. Collectively, our findings demonstrated for the first time that ABHD16A catalyzes the depalmitoylation of IFITM1 to regulate its antiviral activity against HBV, which expands the biological functions of ABHD16A in immune regulation and provides potential targets for HBV infection-related disease therapy.

**IMPORTANCE:**

Nowadays, hepatitis B virus (HBV) infection remains a major global public health problem, with over 375 million people worldwide having been infected. Chronic HBV infection leads to serious liver diseases, such as liver cirrhosis and hepatocellular carcinoma. Therefore, it is urgent to reveal the mechanism of HBV infection and uncover novel drug targets. Interferon and interferon-stimulated genes are responsible for the inhibition of HBV infection. Interferon-inducible transmembrane (IFITM) is distributed on plasma membrane, restricting various virus invasions. Nevertheless, whether and how IFITM regulates HBV infection remains unclear. Here, we show that IFITM1 inhibited the replication of HBV, which depended on palmitoylation modification. In addition, the depalmitoylase α/β-hydrolase domain-containing 16A (ABHD16A) negatively regulates the anti-HBV activity of IFITM1. Overall, our findings provided ABHD16A as a potential target for interfering with HBV replication.

## INTRODUCTION

The hepadnaviridae family hepatitis B virus (HBV) is a 3.2 kb partially double-stranded DNA virus (1). Chronic HBV infection leads to severe liver diseases, including acute hepatitis, fulminant liver failure, chronic hepatitis, cirrhosis, and hepatocellular carcinoma (2). In the past decades, accumulating evidence has indicated that interferons (IFNs) and interferon-stimulated genes (ISGs) inhibit HBV infection, and Type I IFN has been approved to be clinical used for the therapy of chronic hepatitis B (3). However, excessive IFN leads to the production of proinflammatory chemokines, which causes inflammation and tissue damage (4). Thus, it is crucial to discover novel antiviral proteins and identify key factors that can balance the antiviral activity and inflammation response.

Interferon-inducible transmembrane (IFITM) proteins are encoded by ISG and localized on the first line of defense against a variety of microorganisms or pathogen invasion (5). Compared with other IFITM members, IFITM1 is predominantly localized on the plasma membrane and acts as an antiviral restriction factor fighting against non-enveloped and enveloped RNA and DNA viruses (5), including severe acute respiratory syndrome coronavirus (SARS-CoV-2) (6), hepatitis C virus (HCV) (7), human immunodeficiency virus Type I (HIV-1) (8), and Japanese encephalitis virus (JEV) (9). However, current research on IFITM regulating HBV infection is very limited, and the conclusions are unclear. IFITM3 has been reported to interact with HBV receptor NTCP to facilitate virus infection (10). In contrast, a recent study demonstrated that IFITM1 was induced by interleukin-6 family cytokines to inhibit HBV invasion (11). Therefore, it appears important to ascertain whether and how IFITM influences HBV replication.

Protein palmitoylation belongs to post-translational modification (PTM) in which cysteine residues are covalently linked by the fatty acid palmitate (12). Cumulative evidence has indicated that palmitoylation modification affects both host and viral protein functions (13). Moreover, both we and others demonstrated that IFITM1 restricts virus invasion by relying on palmitoylation modification (9, 14, 15). Palmitoylated IFITM1 fusions with the plasma membrane or early endosomes so that the virus is restricted from entering the cytoplasm (16). However, the mechanism of IFITM1 regulation on HBV and whether the mechanism in progress depends on its PTM remained unclear.

The palmitoylation modification is dynamic and reversible; compared with well-established palmitoyl acyltransferase (17), the enzyme capable of catalyzing IFITM depalmitoylation has not been discovered for a long time. α/β-hydrolase domain-containing (ABHD) family members have gradually been reported to be depalmitoylases in recent years (18, 19). To verify whether the ABHD family members regulate the palmitoylation/depalmitoylation modification of IFITM, we screened several ABHD proteins and, for the first time, found that α/β-hydrolase domain-containing 16A (ABHD16A) negatively regulates the palmitoylation and antiviral function of IFITM proteins in humans, swine, and mice (9). Recently, we also discovered that ABHD16A was ubiquitinated by E3 ubiquitin ligase RNF5 (15), which extends the upstream subtle regulatory molecular mechanism of ABHD16A-IFITMs. Nevertheless, we have only conducted several RNA viruses, such as JEV and vesicular stomatitis virus, as models to validate the molecular mechanism of ABHD16A negative regulation of IFITM (9, 15). It is not yet clear whether IFITM1 regulates DNA virus (e.g., HBV) infection mediated by ABHD16A.

In the present work, we first witnessed that HBV infection induced the expression of IFITM1 in blood samples. In addition, overexpression of IFITM1 rather than palmitoylation-defective IFITM1 in HepG2.215 cells could restrict the replication of HBV. These results hinted that the anti-HBV activity of IFITM1 might depend on its palmitoylation modification. What’s more, respectively, knocking out ABHD16A and IFITM1 reduced and augmented HBV replication, which indicated the opposite direction regulation of HBV replication between the two proteins. Overexpression of ABHD16A in wild-type rather than IFITM1 KO HepG2.215 cells increased the replication of HBV, suggesting that ABHD16A facilitated the replication of HBV, which may be achieved by catalyzing the depalmitoylation of IFITM1. Together, these findings reveal a previously unknown view of the IFITM1-mediated antiviral mechanism on HBV, enriching a potential target for therapeutic strategies to prevent and control HBV infection.

## RESULTS

### HBV infection induces the expression of IFITM1

**Fig 1:**
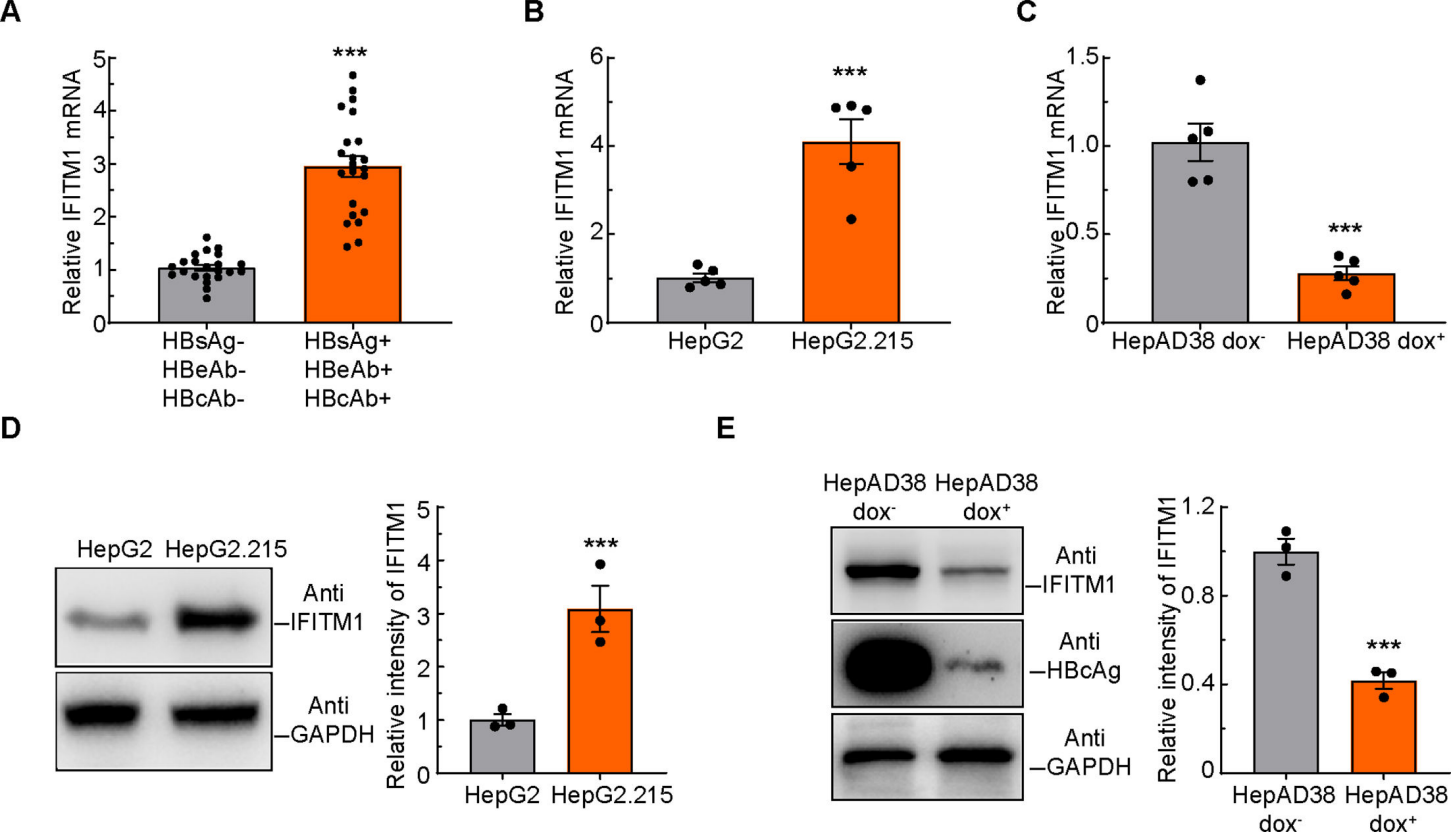
HBV infection induces the expression of IFITM1. (**A**) qRT-PCR of IFITM1 mRNA of blood samples from HBsAg, HBeAb, and HBcAb tested negative and positive patients. *n* = 22 samples were used for quantification for each condition. (**B**) qRT-PCR of IFITM1 mRNA in HepG2 and HepG2.215 cells. (**C**) The mRNA level of HepAD38 intracellular IFITM1 decreased upon treatment with 1 µg/mL Dox for 10 days. (**D**) Western blotting analysis of endogenous IFITM1 amounts in HepG2 and HepG2.215 cells. (**E**) The endogenous IFITM1 protein amount was substantially reduced in response to 1 µg/mL Dox treatment for 10 days in HepAD38 cells. Glyceraldehyde-3-phosphate dehydrogenase (GAPDH) antibody was used to verify equal sample loading. The relative gray values of the bands were calculated by Image J gel analyzer. Data are means ± SEM from three independent experiments. ***P < 0.001 (unpaired two-tailed *t*-test).

To reveal whether IFITM1 could respond to the infection of HBV, we compared the mRNA amount of IFITM1 in blood samples between healthy persons and hepatitis B surface antigen (HBsAg), hepatitis B extracellular antigen antibody (HBeAb), hepatitis B surface antibody (HBcAb) test positive patients according to 2017 European Association for the Study of the Liver Guidelines (20); the results showed significantly increased expression of IFITM1 in HBsAg, HBeAb, and HBcAb test positive patients (Fig. 1A). Furthermore, we used the HBV persistently expressing HepG2.215 cells to verify the expression of IFITM1. By using quantitative real-time PCR (qRT-PCR) and Western blotting assays, we measured IFITM1 mRNA and protein amounts alteration between HepG2 and HepG2.215 cells and found HBV infection induced high levels of IFITM1 expression (Fig. 1B and D). In addition, we applied an HBV-inducible cell line to directly explore the mRNA or protein amount changes of IFITM1 in HepAD38 cells with or without HBV replication. HepAD38 cells were treated with or without 1 µg/mL doxycycline (Dox) for 10 days; cells were, respectively, harvested and subjected to qRT-PCR and Western blotting. The mRNA, as well as protein levels of IFITM1, was significantly decreased after treatment with Dox (Fig. 1C and E). Meanwhile, we assessed the HBcAg protein level and found that with the treatment of Dox, the level of HBcAg dramatically decreased (Fig. 1E). To sum up, HBV infection increased the expression of IFITM1, which hinted that IFITM1 was involved in HBV infection.

### Palmitoylation on conserved cysteines of IFITM1 regulates its anti-HBV activity

**Fig 2:**
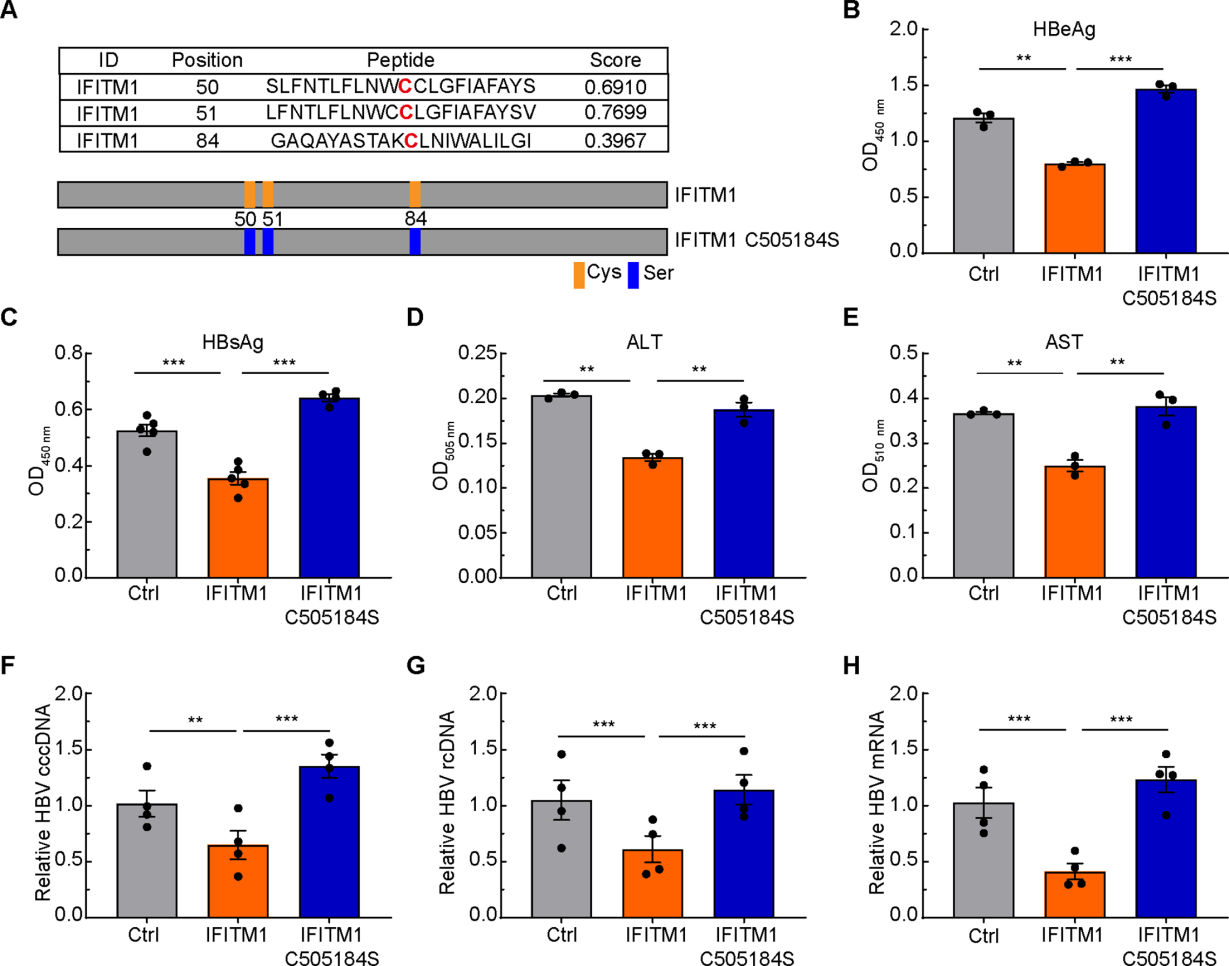
Palmitoylation on conserved cysteines of IFITM1 regulates its anti-HBV activity. (**A**) The palmitoylated residues within IFITM1 were predicted with the GPS-Palm website (http://gpspalm.biocuckoo.cn). (**B–E**) HBeAg (**B**), HBsAg (**C**), ALT (**D**), and AST (**E**) in culture medium of HepG2.215 cells transiently transfected with the indicated plasmids were analyzed. (**F–H**) The HBV cccDNA (**F**), rcDNA (**G**), and mRNA (**H**) in HepG2.215 cells transiently transfected with expression plasmids of pcDNA3.1-Flag, Flag-IFITM1, and Flag-IFITM1 C505184S were calculated by qRT-PCR. Data are means ± SEM from three independent experiments. **P < 0.01; ***P < 0.001 (one-way ANOVA).

Previously, we identified that C50, C51, and C84 residues were conserved cysteines responsible for IFITM1 palmitoylation (Fig. 2A) (21). Thus, we first checked the effect of exogenous expression of full-length and palmitoylation-defective IFITM1 on HBV replication in HepG2.215 cells. Hepatitis B e antigen (HBeAg), HBsAg, glutamic pyruvic transaminase (ALT), and glutamic oxaloacetic transaminase (AST) are well-established indexes for HBV detection, so we estimated them to characterize the regulatory role of IFITM1 on HBV replication. Overexpression of IFITM1 rather than IFITM1 (C505184S) significantly reduced the amount of HBeAg, HBsAg, ALT, and AST (Fig. 2B through E). Biogenesis of covalently closed circular DNA (cccDNA) is composed of nuclear transport, uncoating, repair of relaxed circular double-stranded DNA (rcDNA), and cccDNA chromatinization (22). cccDNA is a transcriptional template in the nucleus. Thus, we examined the intracellular HBV cccDNA, HBV rcDNA, and HBV mRNA of cells transiently expressing full-length and palmitoylation-defective IFITM1 by qRT-PCR, respectively (Fig. 2F through H), and the results showed similar trends as the data of HBeAg, HBsAg, ALT, and AST. Altogether, IFITM1 was shown to conduct as an antiviral factor on HBV, and this function depended on its palmitoylation modification.

### Interaction between ABHD16A and IFITM1 in HepG2.215 cells

**Fig 3:**
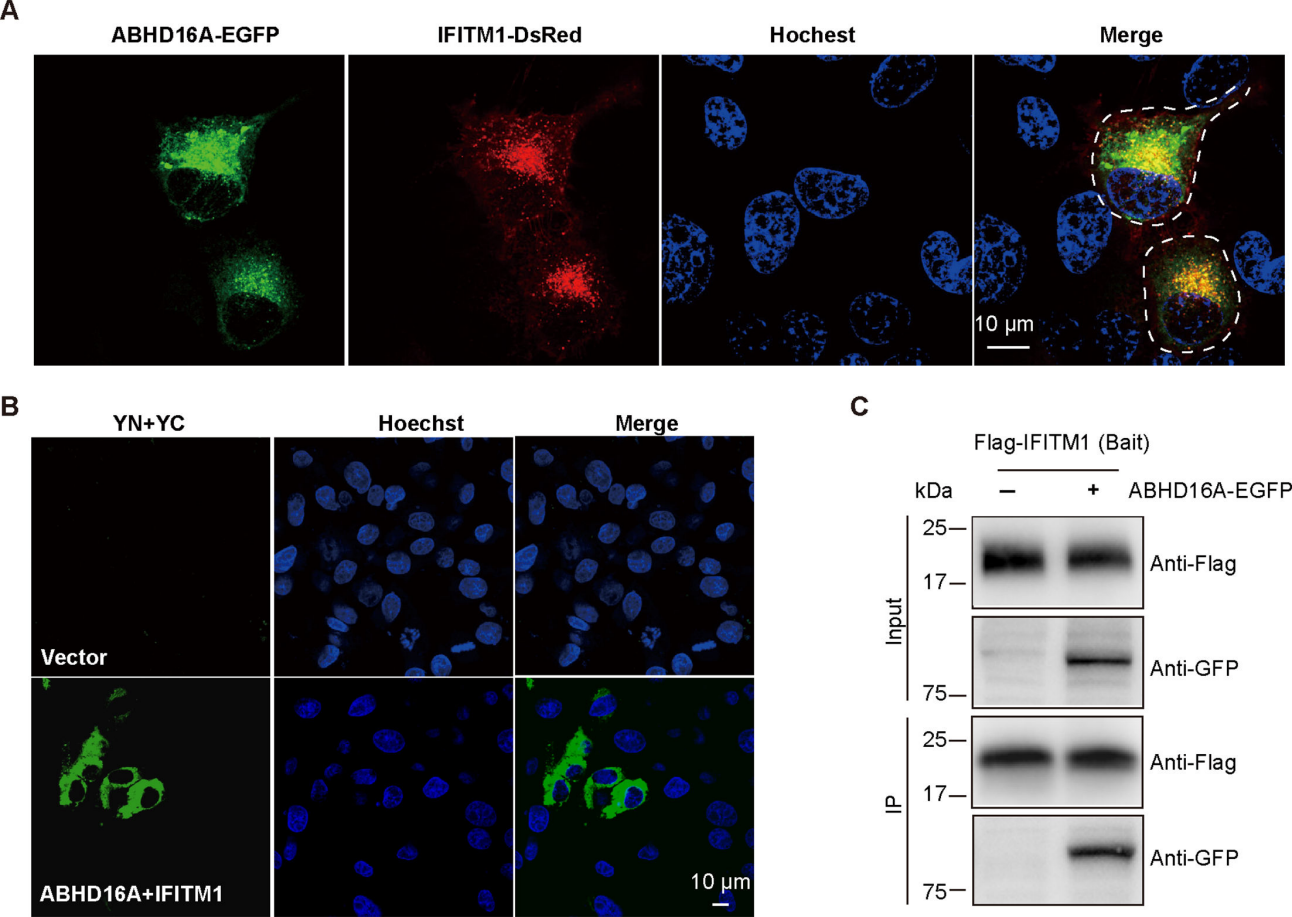
The interaction between ABHD16A and IFITM1 in HepG2.215 cells. (**A**) Subcellular localization of ABHD16A-EGFP with IFITM1-DsRed in HepG2.215 cells. HepG2.215 cells were transfected with expression plasmids of ABHD16A-EGFP and IFITM1-DsRed. Twenty-four hours after transfection, cells were stained with Hoechst for 10 minutes, then washed and fixed with 4% paraformaldehyde. Scale bar: 10 µm. (**B**) Bimolecular fluorescence complementation (BiFC) assay to detect the interaction between IFITM1 and ABHD16A in living cells. Scale bar: 10 µm. (**C**) Co-IP assay to verify the interaction between IFITM1 and ABHD16A. HepG2.215 cells were cotransfected with indicated plasmids for 24 hours, and cell lysates were immunoprecipitated (IP) with anti-Flag antibody. Expression of protein was analyzed by immunoblotting with Flag and GFP antibodies.

Recently, we first reported ABHD16A as a depalmitoylase targeting IFITM proteins and negatively regulating their antiviral activity on JEV (9). The above data proved that the anti-HBV effect of IFITM1 depended on palmitoylation modification, so whether ABHD16A was involved in the process remained to be investigated. Thus, we examined the relationship between ABHD16A and IFITM1 in HepG2.215 cells. We found the colocalization of ABHD16A and IFITM1 via confocal microscopy (Fig. 3A). The results of the bimolecular fluorescence complementation assay in living cells (Fig. 3B) and protein Co-IP assay *in vitro* (Fig. 3C) also verified the interaction between the two proteins.

### ABHD16A regulates HBV replication via depalmitoylation reaction

**Fig 4:**
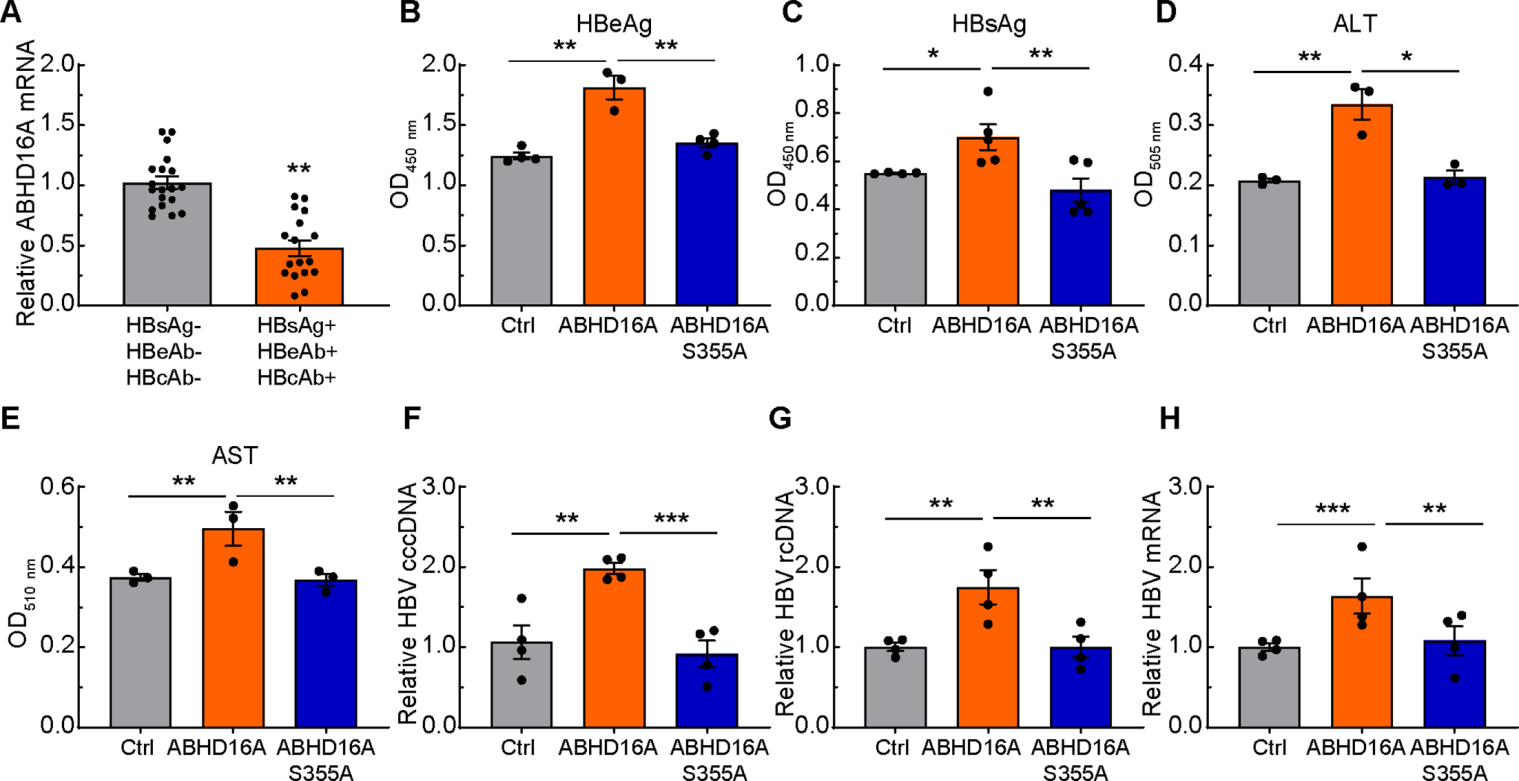
Overexpression of ABHD16A facilitates HBV replication. (**A**) qRT-PCR of ABHD16A mRNA of blood samples from HBsAg, HBeAb, and HBcAb tested negative and positive patients. *n* = 19 blood samples were used for quantification for each condition. (**B–E**) HBeAg (**B**), HBsAg (**C**), ALT (**D**), and AST (**E**) in the culture medium of HepG2.215 cells transiently transfected with indicated plasmids were measured. (**F–H**) HBV cccDNA (**F**), rcDNA (**G**), and mRNA (**H**) in HepG2.215 cells transiently expressing EGFP, ABHD16A-EGFP, and ABHD16A S355A-EGFP were calculated by qRT-PCR. Data are means ± SEM from three independent experiments. *P < 0.05; **P < 0.01; ***P < 0.001 (one-way ANOVA).

Blood samples derived from HBsAg, HBeAb, and HBcAb test positive patients were analyzed to investigate whether HBV infection alters the expression of ABHD16A. By using qRT-PCR, the mRNA amounts of ABHD16A were significantly reduced in response to HBV infection (Fig. 4A), which indicated ABHD16A was involved in the infection of HBV.

To further clarify the specific regulatory role of ABHD16A in HBV infection and whether this regulation relies on palmitoylation PTMs. We mutated Ser at position 355 of ABHD16A to Ala to make the depalmitoylation activation dysfunction (9, 23). The overexpression of ABHD16A in HepG2.215 cells led to an increased amount of HBeAg, HBsAg, ALT, and AST, whereas expression of ABHD16A S355A had no significant effects (Fig. 4B through E). In addition, the HBV cccDNA, HBV rcDNA, and HBV mRNA increased in cells transiently expressed with ABHD16A rather than ABHD16A S355A (Fig. 4F through H). The data above implied that ABHD16A may facilitate the infection of HBV.

**Fig 5:**
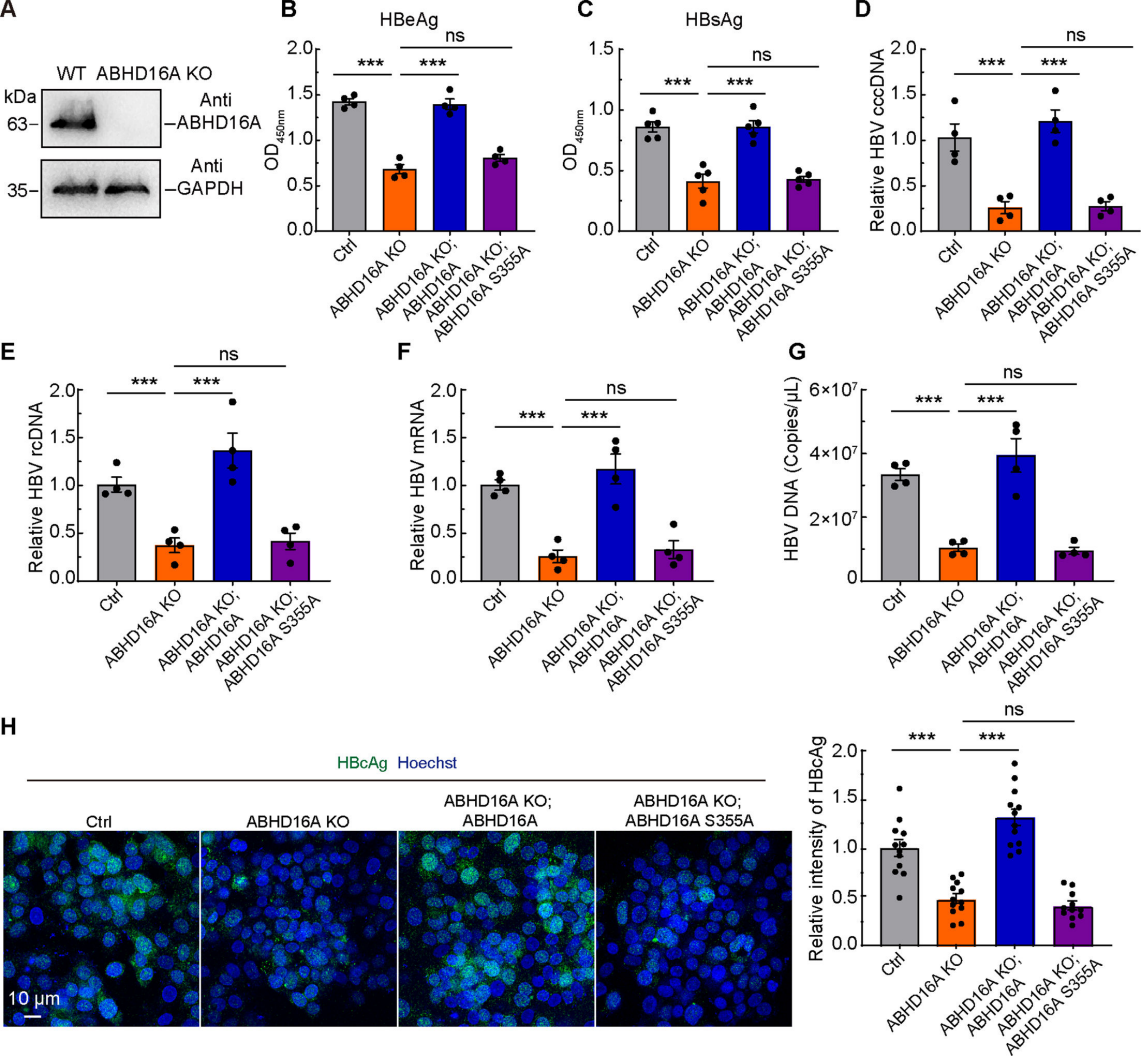
ABHD16A regulates HBV replication via depalmitoylation reaction. (**A**) The amount of ABHD16A protein in HepG2.215 cells and ABHD16A-KO cells was detected by western blotting, GAPDH antibody was used to verify equal sample loading. (**B and C**) HBeAg (**B**) and HBsAg (**C**) in the culture medium of HepG2.215 cells transiently transfected with indicated plasmids were analyzed by ELISA. (**D–G**) HBV cccDNA (**D**), rcDNA (**E**), mRNA (**F**), and DNA (**G**) in wild-type and ABHD16A KO HepG2.215 cells transiently transfected with indicated plasmids were calculated by qRT-PCR. (**H**) Transfected HepG2.215 cells were fixed, immunostained with HBcAg monoclonal antibody, and subjected to confocal microscopy. HA-ABHD16A and HA-ABHD16A S355A expression plasmids were used to carry out rescue experiments. Scale bar: 10 µm. Quantification of the fluorescence intensity of HBcAg for the indicated group. *n* = 11 random regions from three independent dishes. Data are means ± SEM from three independent experiments. ns, no significant difference; ***P < 0.001 (one-way ANOVA).

Considering the reliability of overexpression experimental data, we constructed ABHD16A KO HepG2.215 cells via the CRISPR/Cas9 system to confirm the involvement of ABHD16A in HBV replication (Fig. 5A). We carried out Western blotting, microscopic images, and CCK8 assays to check whether the cells were available for investigation. The results showed that there was no expression of ABHD16A in ABHD16A KO HepG2.215 cells, in which both the cell morphology and cell proliferation remained in a normal state (Fig. 5A; Fig. S1A and B). Knockout of ABHD16A resulted in decreased HBeAg, HBsAg, HBV cccDNA, rcDNA, mRNA, and DNA levels, and the reduction could be rescued by transiently expressed ABHD16A but not ABHD16A S355A (Fig. 5B through G). Furthermore, we detected the distribution and expression of intracellular HBV antigen (e.g., HBcAg). By using immunofluorescence, HBcAg signals were detected in cell nuclei and cytoplasm (Fig. 5H). In ABHD16A KO HepG2.215 cells, the fluorescence intensity was significantly decreased when compared with WT cells. However, the decreased level of viral antigen signals was successfully rescued by the expression of HA-ABHD16A rather than the depalmitoylation reaction defective HA-ABHD16A S355A (Fig. 5H). Above all, ABHD16A facilitates the replication of HBV in HepG2.215 cells.

### Depalmitoylase ABHD16A inhibits the anti-HBV activity of IFITM1

**Fig 6:**
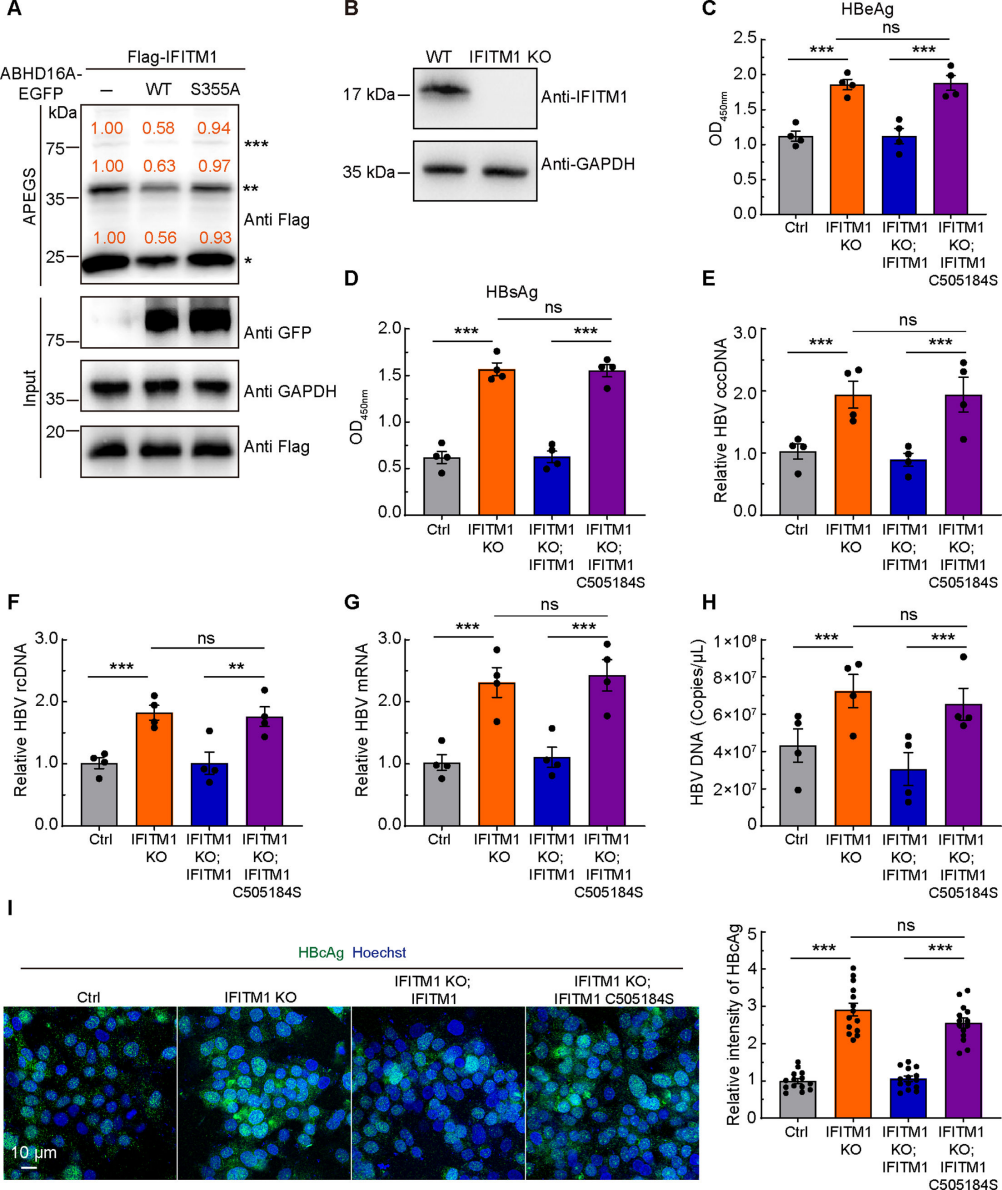
Depalmitoylase ABHD16A inhibits the anti-HBV activity of IFITM1. (**A**) APEGS assay to verify the depalmitoylation site of ABHD16A on IFITM1. The number of PEGylation exchanges is indicated by asterisks. The relative gray values of the bands (palmitoylated/Input) were calculated by the Image J gel analyzer. (**B**) The expression of endogenous IFITM1 protein in wild-type and IFITM1-KO HepG2.215 cells was detected by Western blotting. (**C and D**) ELISA analysis of HBeAg (**C**) and HBsAg (**D**) in culture medium of HepG2.215 cells transiently transfected with indicated plasmids. (**E–G**) HBV cccDNA (**E**), rcDNA (**F**), and mRNA (**G**) in wild-type and IFITM1 KO HepG2.215 cells transiently transfected with indicated plasmids were analyzed by qRT-PCR. (**H**) HBV DNA in the culture medium of wild-type and IFITM1 KO HepG2.215 cells transiently transfected with indicated plasmids was analyzed by qRT-PCR. (**I**) Transfected wild-type or IFITM1 knockout HepG2.215 cells were fixed, immunostained with HBcAg monoclonal antibody, and subjected to confocal microscopy. Flag-IFITM1 and Flag-IFITM1 C505184S expression plasmids were used to carry out rescue experiments. Scale bar: 10 µm. Quantification of the fluorescence intensity of HBcAg for the indicated group. *n* = 11 random regions from three independent dishes. Data are means ± SEM from three independent experiments. ns, no significant difference; *P < 0.05; **P < 0.01; ***, *P* < 0.001 (one-way ANOVA).

The weak anti-HBV effects in cells transiently expressing IFITM1 C505184S indicated that the antiviral activity of IFITM1 on HBV depended on palmitoylation modification (Fig. 2); together with the confirmation that ABHD16A interacts with IFITM1 (Fig. 3) and regulates the infection of HBV (Fig. 4 and 5), we speculated that ABHD16A may regulate the process of IFITM1’s resistance to HBV infection through depalmitoylation. To verify this, we carried out an acyl-PEGyl exchange gel-shift (APEGS) assay, which is widely used to label palmitoylated residues with methoxy polyethylene glycol maleimide (mPEG-Mal) (13, 21). By using the APEGS assay, we demonstrated that the expression of full-length ABHD16A rather than ABHD16A S355A in HepG2.215 cells decreased the mPEG-Mal-linking bands of IFITM1, indicating that ABHD16A catalyzed the depalmitoylation reaction on IFITM1 and S355 is the critical site for depalmitoylase activity of ABHD16A in HepG2.215 cells (Fig. 6A).

To dissect the anti-HBV mechanism of IFITM1, we constructed IFITM1 knockout (KO) HepG2.215 cells through the CRISPR/Cas9 system. Western blotting, microscopic, and CCK8 assays were used to confirm the usability of IFITM1 KO HepG2.215 cells (Fig. 6B; Fig. S1C and D). Then we explored the secreted HBV antigens and intracellular HBV cccDNA, HBV rcDNA, and HBV mRNA levels in wild-type and IFITM1 KO HepG2.215 cells transiently expressing IFITM1 and IFITM1 C505184S. Loss of IFITM1 led to elevated HBeAg, HBsAg, HBV cccDNA, rcDNA, mRNA, and DNA levels, and the phenotype could be rescued by transiently expressed IFITM1, whereas IFITM1 C505184S could not (Fig. 6C through H). Moreover, we investigated the impact of IFITM1 on the intracellular HBcAg. We witnessed that the loss of IFITM1 promotes HBV replication (Fig. 6I). In contrast, overexpression of an IFITM1 construct lacking all three palmitoylated cysteines (C505184S) failed to restore HBcAg intensity (Fig. 6I), suggesting that the anti-HBV function of IFITM1 depends on its palmitoylation modification.

**Fig 7:**
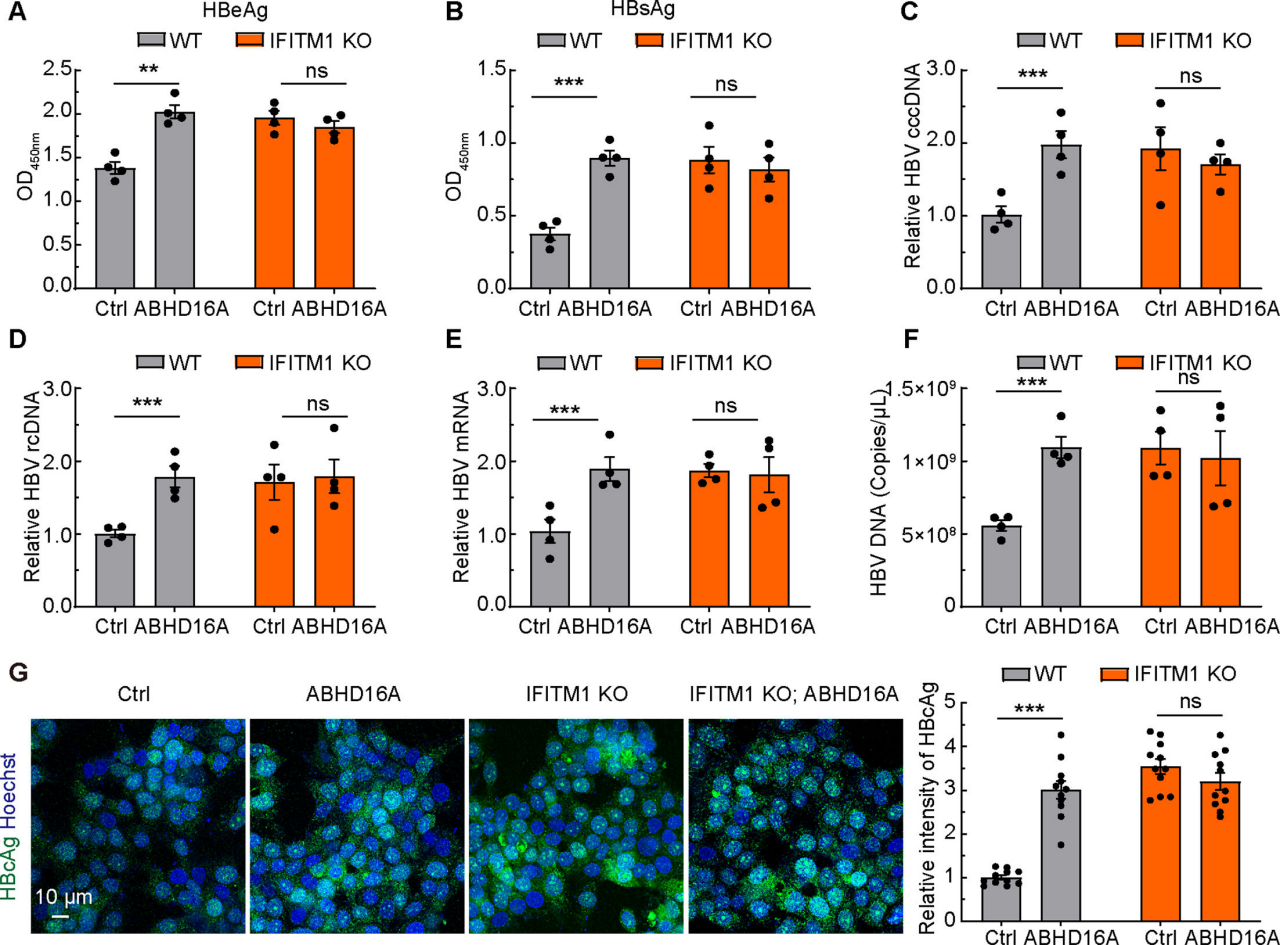
ABHD16A catalyzed the depalmitoylation modification on IFITM1, which inhibited the anti-HBV function of IFITM1. (**A and B**) ELISA analysis of HBeAg (**A**) and HBsAg (**B**) in culture medium of HepG2.215 cells transiently transfected with indicated plasmids. (**C–E**) The level of HBV cccDNA (**C**), rcDNA (**D**), and mRNA (**E**) in HepG2.215 cells and IFITM1-KO HepG2.215 cells transiently expressing EGFP and ABHD16A-EGFP, respectively. (**F**) HBV DNA amounts in the culture medium of HepG2.215 cells and IFITM1-KO HepG2.215 cells transiently expressing EGFP and ABHD16A-EGFP, respectively. (**G**) Transfected wild-type or IFITM1 knockout HepG2.215 cells were fixed, immunostained with HBcAg monoclonal antibody, and subjected to confocal microscopy. Scale bar: 10 µm. Quantification of the fluorescence intensity of HBcAg for the indicated group. *n* = 11 random regions from three independent dishes. Data are means ± SEM from three independent experiments. ns, no significant difference; **P < 0.01; ***P < 0.001 (one-way ANOVA).

Next, we sought to dissect whether ABHD16A regulates the anti-HBV infection of IFITM1 via depalmitoylation modification. We assessed the intracellular HBV cccDNA, HBV rcDNA, HBV mRNA, HBcAg, and extracellular HBeAg, HBsAg, HBV DNA level of the cultural medium in wild-type and IFITM1 KO HepG2.215 cells transiently transfected with EGFP-N1 and ABHD16A-EGFP expression plasmids, respectively. The results show that transiently expressed ABHD16A in HepG2.215 cells could augment HBV replication, but transiently expressed ABHD16A in IFITM1 KO HepG2.215 cells could not (Fig. 7). These data indicated that ABHD16A catalyzed the depalmitoylation modification on IFITM1, which inhibited the anti-HBV function of IFITM1.

### The ABHD16A inhibitor KC01 suppresses the replication of HBV in HepG2.215 cells

**Fig 8:**
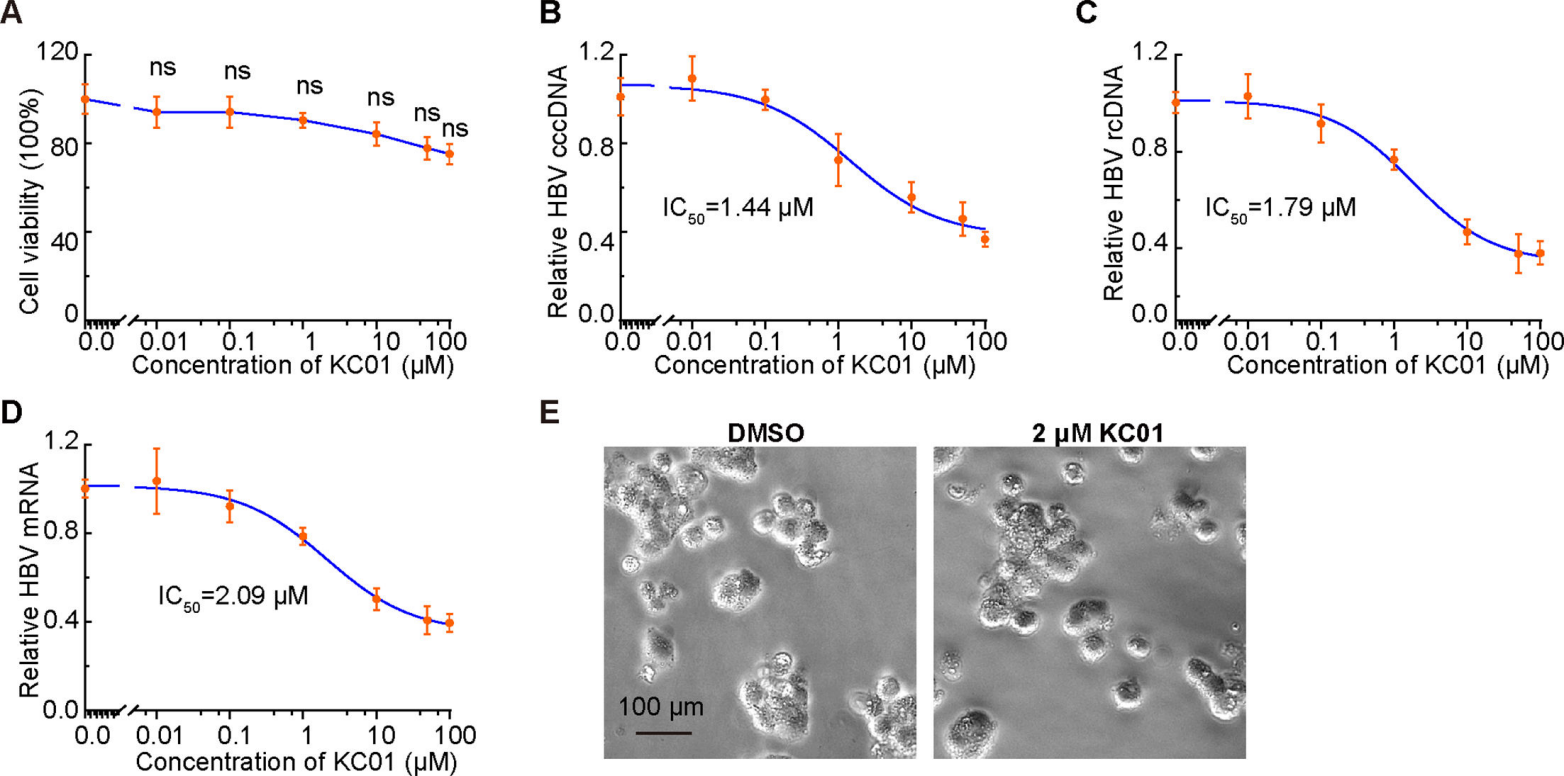
The ABHD16A inhibitor KC01 suppresses the replication of HBV in HepG2.215 cells. (**A**) Quantification of the cell viability treated with the indicated concentration of KC01 for 24 hours. (**B–D**) Quantification of the intracellular cccDNA, rcDNA, and mRNA amounts of HBV treated with increasing concentrations of KC01 for 24 hours. IC50 was calculated using nonlinear regression analysis from three independent experiments. (**E**) Phase contrast imaging reveals the morphology of HepG2.215 cells treated with DMSO or 2 µM KC01 for 24 h. Scale Bar: 100 µm. Data are means ± SEM from three independent experiments. ns, no significant difference (one-way ANOVA).

Since the novel regulatory role of ABHD16A in HBV replication, we sought to validate whether treatment with the ABHD16A inhibitor affects the replication of HBV. Kamat et al. have screened several compounds and demonstrated KC01 as the most potent inhibitor targeting ABHD16A (24). Previously, we have applied KC01 in swine PK15 cells and found the JEV mRNA amount significantly reduced upon KC01 treatment (9). Here, we carried out the KC01 treatment assay on HepG2.215 cells and set out to calculate the half-maximal inhibitory concentration (IC50) of KC01. Six concentration gradients of KC01 were tested for their anti-HBV activities, and all concentrations showed no significant cytotoxicity effect on cells until 100 µM for 24 hours (Fig. 8A). Further analyses showed that KC01 was fairly potent in restricting HBV replication (IC50  =  1.44 µM for HBV cccDNA; IC50  =  1.79 µM for HBV rcDNA; IC50  =  2.09 µM for HBV mRNA) (Fig. 8B through D). Finally, we treated HepG2.215 cells with 2 µM KC01 for 24 hours and found that KC01 does not affect cell morphology (Fig. 8E). Together, these results suggested that ABHD16A inhibition (e.g., by KC01) can potently restrict the HBV infection in cells.

## DISCUSSION

HBV infection induced chronic hepatitis B, liver cirrhosis, and hepatocellular carcinoma, which caused significant challenges for healthcare services worldwide (2). IFN combined with other drugs has been applied for chronic hepatitis B therapy (25, 26). Palmitoylated IFITM proteins can be induced by IFN to confer various virus infections (5, 13). However, whether and how IFITMs restrict HBV infection remained largely elusive. In the present study, we identified that palmitoylated IFITM1 counteracted the replication of HBV. Furthermore, the newly found depalmitoylase ABHD16A interacts with IFITM1 and downregulates the palmitoylation level of IFITM1, which attenuates the anti-HBV activity of host cells. These results uncovered the novel role of palmitoylation modification in host-HBV interaction.

Although the genomic sequence ABHD16A is located within the human major histocompatibility complex Class III region (23), the regulatory role of ABHD16A in innate immunity, especially in viral infection, has not been thoroughly investigated for a long time. In recent years, we and other research groups have successively discovered ABHD16A and other ABHD family members (e.g., ABHD10 and ABHD17A) as novel depalmitoylases (9, 15, 18, 19, 27). Combined with the dynamic palmitoylation/depalmitoylation modification playing key roles in host-virus interaction (13), we decided to verify whether and how ABHD16A regulates virus infection. In 2022, we demonstrated that ABHD16A targeted IFITM1 for depalmitoylation, which downregulated the anti-RNA virus effects of IFITM1 (9). Subsequently, taking advantage of the bioinformatics technique, we revealed that E3 ubiquitin ligase RNF5 negatively regulates ABHD16A, which weakens the depalmitoylation effect of ABHD16A on IFITM1 and consequently restores the antiviral activities of host (15). Herein, we demonstrated for the first time the key regulatory role of ABHD16A-IFITM1 interaction in HBV infection, which expanded the function of ABHD16A in the innate immune system and provided a new target for the treatment of chronic hepatitis B.

IFITM1–IFITM3 have been shown to function as inhibitors of HCV entry (7). However, our understanding of the regulatory role of IFITM in HBV infection remains limited and unclear. A recent study focused on the JAK-STAT signaling pathway showed that interleukin-6 family cytokines oncostatin M (OSM) inhibited HBV replication via IFITM1 (11). In contrast, IFITM3 is reported to be not restricting, but supporting HBV/ hepatitis D virus (HDV) infection by facilitating viral entry through interaction with viral receptor NTCP (10). Moreover, IFITM2 is shown to inhibit IFN alpha pathway activation and block the anti-HBV efficacy of exogenous IFN via transportation to dendritic cells (28). In fact, cumulative evidence has indicated that IFITM proteins play multiple regulatory roles during viral infection. Recent studies show that IFITM proteins were hijacked by SARS-CoV-2, which facilitates virus infection (29). Furthermore, Shi et al. decreased the amphipathicity of the helix within IFITM3, which resulted in IFITM3 becoming viral infection enhancers (6). In the present study, we uncovered the increased expression of IFITM1 in HBV-infected patients, and by using the well-established HepG2.215 cells, we demonstrated the anti-HBV replication activity of palmitoylated IFITM1. However, how IFITM1 interacts with HBV proteins and whether other IFITM proteins, such as IFITM2, IFITM3, IFITM5, and IFITM10, play important roles in HBV infection need to be further studied.

As our data shown in Fig. 1, IFITM1 expression in HepG2.215 cells, which are a human liver cancer cell line that can express HBV antigen and secrete intact HBV particles, showed the same ascension as the result of HBsAg, HBeAb, and HBcAb test-positive patients, who are HBV carriers. Nevertheless, Li et al. demonstrated that HBV core protein is reported to inhibit IFITM1 by interacting with BRG1/hBRM-associated factors 200 (BAF200) (30). This conclusion differs from our present results. How could this happen? First, the modes of HBV infection are different. HBV in our work is derived from the integrated gene expression of HepG2.215 cells rather than the supernatant of HepAD38 cells. Second, host cells may respond differently to a variety of concentrations and forms of viruses, which leads to divergent levels of IFITM1. The present data about IFITM1 expression in HBV-infected cells and blood samples in our study supported that the increased expression of IFITM1 restricted HBV replication. Based on this, we think that in the future, it will be helpful to further verify whether the mRNA or protein level of IFITM1 in the liver samples was affected by HBV infection and consider that IFITM1 might be a marker for predicting the response to HBV infection.

Due to the use of HepG2.215 and HepAD38 cells that integrated with the HBV genome in this study, rather than adding HBV particles to the cell culture medium to carry out infection experiments, we are currently unable to reveal the specific sites and time points at which ABHD16A and IFITM1 exert antiviral effects. IFITM1 is localized mostly on the plasma membrane, and we found that ABHD16A inhibits the localization of IFITM1 on the plasma membrane through depalmitoylation in a previous study (9, 31). Therefore, we speculated that ABHD16A-IFITM1 may play roles in resisting virus invasion into cells or in the process of virus egress. In addition, Ye et al. recently reported that IFITM1 is involved in OSM-mediated anti-HBV activity, which may be achieved through 2′−5′-oligoadenylate synthetase antiviral response and ISG15 antiviral mechanism (11). The specific molecular mechanism requires further research.

It is widely known that HBV is not cytotoxic, while hepatocellular injury and HBV pathogenesis are caused by the host immune responses in infected hepatocytes. It is reported that HBV replication and host immune responses determine the clinical symptoms of chronic hepatitis B patients (32). HBV can escape the host response through signal interference, effector modulation, and continual viral genetic variation (33). In addition, IFITM proteins can prevent the fusion of the viral and cellular membranes by modulating membrane rigidity and curvature (34–38). Hereby, as the cell’s first line of antiviral defense, the innate immune pathway is vital to HBV infection. The HBV life cycle is a complicated process composed of five steps: viral entry, cccDNA biogenesis, progeny nucleocapsid production, virion formation, and viral egress (39), so the research and treatment of HBV pose certain difficulties. Considering the clinical relevance of our data, further research needs to explore whether the exogenous addition of IFITM1 or ABHD16A inhibitor KC01 can inhibit HBV infection at both cellular and clinical levels.

In conclusion, our study demonstrated that IFITM1 acts as an antiviral factor on HBV, and this effect relies on palmitoylation modification. ABHD16A interacted and catalyzed the depalmitoylation of IFITM1, which downregulated the anti-HBV effects of IFITM1. Our findings provide new insights into the mechanisms of host-HBV interaction and provide potential therapeutic strategies for liver diseases.

## MATERIALS AND METHODS

### Study subjects

According to the 2017 European Association for the Study of the Liver Guidelines (20), patients’ blood samples were classified into HBsAg, HBeAb, and HBcAb test-positive and negative groups. Pregnant persons and patients with HCV, HDV, or HIV infection were not within the scope of testing.

### Plasmid construction

The cDNAs of IFITM1 (Gene ID: 8519) and ABHD16A (Gene ID: 7920) were amplified from the isolated total RNA of *Homo sapiens embryonic* kidney HepG2 cells. To generate EGFP-, DsRed-, HA-, and Flag-tagged ABHD16A and IFITM1 plasmids, the cDNA fragments were digested with restriction endonuclease and individually cloned into the same sites of pEGFP-N1, pDsRed-monomer-N1, pcDNA3.1(+)-HA, and pcDNA3.1(+)−3 × Flag, respectively. The S355A mutant of ABHD16A and the C505184S mutant of IFITM1 were constructed by the overlap extension method (40).

### Cell culture and transfection

HepG2 (human hepatoma cells), HepG2.215 (human hepatoma cells with HBV), and HepAD38 cells were cultured in Dulbecco’s modified Eagle’s medium containing 10% fetal calf serum, 100 mg/mL streptomycin, 100 U/mL penicillin, and 4 mM L-glutamine at 37°C in a 5% CO_2_ atmosphere. 1 µg/mL Dox was added to HepAD38 cells for 10 days to generate HepAD38 cells without HBV. For cell transfection, 5 × 10^5^ cells were seeded into six-well plates with 2 mL culture medium, replaced with a new culture medium after 24 hours of cell cultivation. 2.5 µg expression plasmid carrying target genes were introduced into cells using Lipofectamine 3000 (#L3000001; Invitrogen, USA) according to the manufacturer’s instruction using a 3:1 Lipofectamine to DNA ratio. A fluorescence microscope was used to evaluate the transfection efficiency. Cell proliferation was measured with an Enhanced CCK8. 2 × 10^4^ cells were seeded into 96-well plates with 100 µL of culture medium per well. After sufficient cell adhesion, 10 µL of CCK-8 agent was added to the wells and incubated at 37°C for 2 hours. Place the plate on the microplate reader to measure the absorbance value at 450 nm.

### Western blotting

To determine the expression of cellular proteins, HepG2.215 cells were transfected with target genes in six-well plates. After 24 hours of cultivation, cells were washed twice with cold PBS. Then using 1 mL of radioimmunoprecipitation assay (50 mM Tris pH 7.4, 150 mM NaCl, 1% Triton X-100, 1% sodium deoxycholate, and 0.1% SDS) lysis buffer (#P0013; Beyotime; China) supplemented with 1 mM protease inhibitor cocktail (#P001; NCM Biotech; China), 1 mM phenylmethylsulfonyl fluoride (PMSF), 10 mM dithiothreitol (DTT), 40 mg/mL DNase I, 1 mg/mL leupeptin, pepstatin to harvest cells. Before accumulating the protein sample, the cells need to be lysed on ice for 20 minutes. To remove cell debris, samples were centrifuged for 30 minutes at 4°C, 12,000 *g*. Cell lysates were mixed with 1 × SDS PAGE loading buffer (#WB2001; NCM Biotech; China), heated at 100°C for 10 minutes, and separated on SDS-PAGE at a constant voltage of 110 V for 40 minutes, blotted onto 0.45-mm-pore polyvinylidene difluoride (#IPVH00010; Millipore, Germany) membranes at a constant current of 200 mA for 120 minutes. After the transfer, the membrane was blocked in 5% BSA for 1 hour at room temperature. Proteins were incubated using primary antibodies against GFP (#66002–1; Proteintech, USA), Flag (#F3165; Sigma-Aldrich, USA), GAPDH (#10494–1; Proteintech, USA), IFITM1 (#ab233545; Abcam, USA), ABHD16A (#SRP08788; Saierbio, China), HBcAg (#ab316283; Abcam, USA) at 4°C overnight. After three washing steps in Tris-buffered saline with Tween 20 (TBST) (20 mM Tris, 137 mM NaCl, pH 7.6, 20% Tween-20), the membranes were incubated with horseradish peroxidase-conjugated secondary antibodies (Proteintech; USA) at room temperature for 1 hour. At last, using an ultra-sensitive electrochemiluminescence (ECL) kit (Beyotime; China), membranes were scanned by a chemiluminescence imaging instrument, and band intensities were quantified by Image J.

### Co-immunoprecipitation (Co-IP)

Transfected HepG2.215 cells were washed twice with phosphate buffered saline (PBS) and harvested by using immune coprecipitation with 100 µL lysis buffer (20 mM Tris 7.5, 150 mM NaCl, 1% Triton X-100, 1% sodium deoxycholate, and 0.1% SDS supplemented with 10 mM DTT, 1 mM PMSF, 40 mg/mL DNase I and 1 mg/mL leupeptin, pepstatin, and aprotinin) for each well. Then the cell lysate was centrifuged at 4°C, 12,000 rpm for 15 minutes. Protein A + G magnetic beads (#P2108; Beyotime, China) binding with Flag antibody were added to the reaction system and incubated at 4°C for 12 hours to immunoprecipitate Flag-IFITM1. Afterward, the magnetic beads were washed with PBS three times and boiled in 1 × SDS PAGE loading buffer for 5 min. Finally, the supernatant was subjected to SDS-PAGE followed by Western blotting.

### Confocal microscopy

HepG2.215 cells were cotransfected with expression plasmids of ABHD16A-EGFP and IFITM1-DsRed. After cultivating for 24 hours, washed cells twice with preheated PBS, added 500 µL of 4% paraformaldehyde (PFA) to fix cells at room temperature for 15–20 minutes. Hoechst 33342 (#P0133; Beyotime, China) was used to stain the nuclei. A Leica TCS SP8 laser scanning confocal microscope with a Nikon Plan Apo × 60/1.5 oil lens objective was used to capture fluorescent signals.

### Bimolecular fluorescence complementation assay (BiFC)

The cDNA fragments of IFITM1 and ABHD16A were fused with YN (aa 1–155 of yellow fluorescent protein [YFP]) and YC (aa 156–239 of YFP), respectively (41). HepG2.215 cells were transiently transfected with the following plasmids: YN+YC, or IFITM1-YN+ABHD16A-YC. 24 hours later, Hoechst 33342 was used to stain cells for 10 minutes and then analyzed by confocal microscope. The interaction between ABHD16A and IFITM1 in living cells was denoted by YFP signals.

### Immunofluorescence (IF)

HepG2.215 cells grown on 10 µg/mL fibronectin (#F2006, Merck, USA)-coated 35 mm glass-bottomed dishes were fixed with 4% PFA for 20 minutes. Then cells were blocked and permeabilized with 5% BSA and 0.5% Triton-X100, respectively. The HBcAg primary antibody (dilution: 1:200; #ab316283; Abcam, USA) and CoraLite-488-conjugated Goat-anti-Rabbit secondary antibody (dilution: 1:800; #RGAR002; Proteintech, USA) were used to detect intracellular HBcAg. Afterward, coverslips were mounted in an antifade mounting medium with Hoechst 33342 (#P0133; Beyotime, China) for fluorescence imaging. Images were acquired using a Leica TCS SP8 laser scanning confocal microscope equipped with a Nikon Plan Apo × 60/1.5 oil lens objective.

### Enzyme-linked immunosorbent assay (ELISA) of secreted HBeAg and HBsAg

The HBeAg and HBsAg in HepG2.215 cell culture medium were detected by kits of HBeAg (MM-51632H2, MeiMian, China) and HBsAg (MM-62949H1, MeiMian, China), respectively. The absorption value was obtained through a microplate reader at OD 450 nm.

### Secreted ALT and AST detection

The secreted ALT and AST in HepG2.215 cell culture medium were detected by kits of ALT (C009-2-1, JianCheng Bioengineering Institute, China) and AST (C010-2-1, JianCheng Bioengineering Institute, China). The ALT and AST absorption values were obtained through a microplate reader at OD 505 nm and OD 510 nm, respectively.

### Quantitative real-time PCR (qRT-PCR)

The total RNA in HepG2.215 cells was extracted by RNA-easy isolation reagent (#R701-02-AA, Vazyme, China) according to the instructions. The extracted RNAs were reverse transcribed to cDNA using SuperScript (#18064071; Invitrogen, USA) with oligo (dT) primers and a gDNA wiper to eliminate traces of genomic DNA. qRT-PCR reactions were carried out by using the StepOne Plus Real-Time PCR System (Applied Biosystems, USA) with the 2 × HQ SYBR qPCR kit (#ZF503-2, ZomanBio, China). The relative expression of target genes was quantified using the 2^−ΔΔCt^ method. Each experimental transcript was tested in triplicate. Primers used were listed below: (5′−3′): IFITM1: forward: ATCCTGTTACTGGTATTCGG; reverse: TATAAACTGCTGTATCTAGG, ABHD16A: forward: GATACGTACTATCAGCCCCGTG; reverse: AGGCGAAGGGAGAGGAGTAAT, HBV mRNA: forward: ACCTCTGCCTAATCATCTC; reverse: GTAAGACAGGAAATGTGAAAC, HBV DNA: forward: GTTGCCCGTTTGTCCTCTAATTC; reverse: GGAGGGATACATAGAGGTTCCTT, HBV rcDNA: forward: GGAGGGATACATAGAGGTTCCTTGA; reverse: GTTGCCCGTTTGTCCTCTAATTC, HBV cccDNA: forward: CCCCGTCTGTGCCTTCTC; reverse: CAGCTTGGAGGCTTGAACAGT.

### HBV DNA, rcDNA, and cccDNA extraction and detection

The extracellular HBV DNA in HepG2.215 cell culture medium and intracellular HBV DNA in HepG2.215 cells were extracted by the Genomic DNA Purification Kit (#2868751, Thermo Scientific, USA). Mung bean nuclease (#M0250S, NEB, USA), which catalyzes the removal of single-stranded extension in double-stranded DNA, could digest the extracted intracellular DNA to cccDNA. The HBV DNA, rcDNA, and cccDNA were amplified and measured by qRT-PCR.

### Construction of IFITM1 KO and ABHD16A KO HepG2.215 cells

The CRISPR/Cas9 system was used to construct the IFITM1 KO and ABHD16A KO cell lines. The primers of CRISPR/Cas9 guide RNA (gRNA) targeting the human *ifitm1* and *abhd16a* genes were designed by using http://crispr-era.stanford.edu/index.jsp. The *ifitm1* primer sequences of sgRNA are: forward, 5′-CACCGTGATCACGGTGGACCTTGGA-3′; reverse, 5′- AAACTCCAAGGTCCACCGTGATCAC-3′. The *abhd16a* primer of sgRNA sequences are: forward, 5′-CACCGGTACTATCAGCCCCGTGCCC-3′; reverse, 5′- AAACGGGCACGGGGCTGATAGTACC-3′. The plasmids of LentiCRISPRV2-sgRNA-*ifitm1* and LentiCRISPRV2-sgRNA-*abhd16a* were transfected into HepG2.215 cells and screened with 2 µg/mL puromycin. The IFITM1 KO and ABHD16A KO cells were obtained using the limiting dilution culture method. Ultimately, the DNA and protein were, respectively, extracted for PCR and SDS-PAGE analysis to identify whether the gene knockout was successful.

### Acyl-PEGyl exchange gel-shift assay (APEGS)

Percher et al. (42) and Kanadome et al. (43) recounted the APEGS method, which our laboratory optimized as reported before (9, 31). HepG2.215 cells were transfected with Flag-IFITM1 expression plasmids for 24 hours. Cells were lysed with lysis buffer (50 mM triethanolamine (TEA), 150 mM NaCl, 4% SDS, 5 mM EDTA, and 1 µg/mL leupeptin, pepstatin, and aprotinin, pH 7.3). Cell lysate was incubated with 200 mM tris-(2-carboxyethyl) phosphine at 55°C for 1 hour and 1 M N-ethyl maleimide at 25°C for 4 hours. Chloroform-methanol precipitation (Methanol:Chloroform:H_2_O = 4:1.5:3) was used twice to recover proteins. TEA buffer was used to resuspend it. The mixtures described above continued to be incubated with 0.75 M NH_2_OH at 25°C for 4 hours. Maleimide-conjugated PEGs (mPEG-mal, 5  kDa) in the TEA were used to replace the S-palmitate of cysteines at 25°C for 4 hours. The palmitoylation blots of all test samples were detected with monoclonal anti-FLAG M2 (#F3165; Sigma-Aldrich, USA) through western blotting. The gray values of all palmitoylated IFITM1 bands to input IFITM1 bands were considered as the palmitoylated level.

### Drug treatment

The ABHD16A inhibitor KC01 (#GC12789; GlpBio, USA) was used at 2 µM and treated for 24 h.

### Statistical analysis

All data are presented as the means ± SEM. An unpaired two-tailed *t*-test was used to determine significant differences between two groups, and a one-way analysis of variance (ANOVA) followed by Tukey’s *post hoc* test was used to evaluate differences between three or more groups. Statistical significance was set as *P < 0.05; **P < 0.01; ***P < 0.001; ns, not significant. All analyses were performed using GraphPad Prism 9 (GraphPad Software, CA, USA).

## Data Availability

The data that support the findings of this study are available from the corresponding author upon reasonable request.

## SUPPLEMENTAL MATERIAL

Fig. S1 spectrum.03095-24-s0001.docx Proliferation of ABHD16A KO and IFITM1 KO HepG2.215 cells.

ASM does not own the copyrights to Supplemental Material that may be linked to, or accessed through, an article. The authors have granted ASM a non-exclusive, world-wide license to publish the Supplemental Material files. Please contact the corresponding author directly for reuse.
