# Supplementary material for: Depalmitoylase ABHD16A negatively regulates the anti-hepatitis B virus activity of IFITM1
Source: Microbiol Spectr. 2025 May 28;13(7):e03095-24. doi: 10.1128/spectrum.03095-24 (PMC12210952; doi:10.1128/spectrum.03095-24)
Supplement: Fig. S1 — Proliferation of ABHD16A KO and IFITM1 KO HepG2.215 cells. [file spectrum.03095-24-s0001.docx]

**Supplemental Material**

**
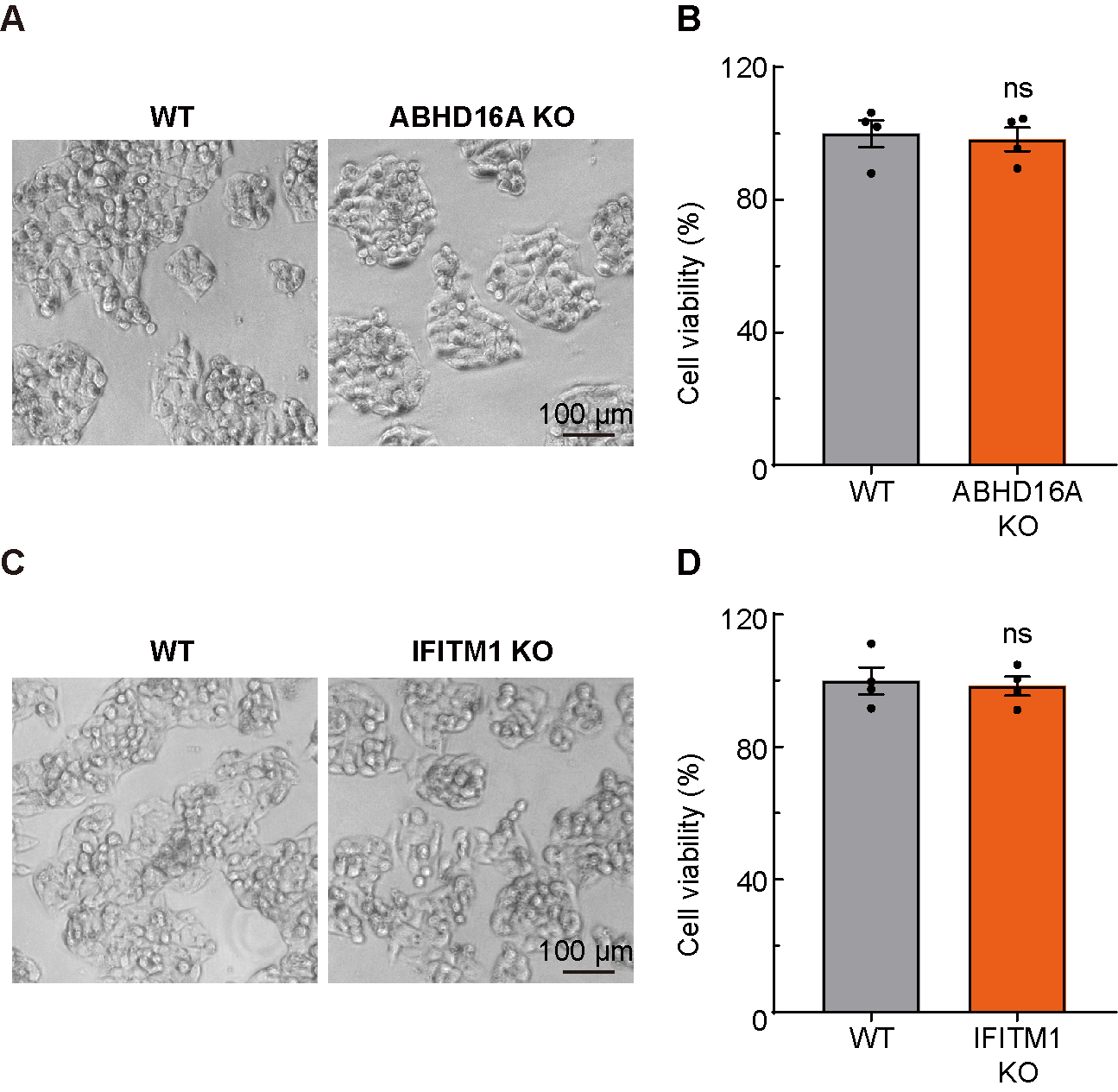
**

**Figure S1. Proliferation of ABHD16A KO and IFITM1 KO HepG2.215 cells.** (A) Phase contrast imaging reveals the morphology of wild-type and ABHD16A KO HepG2.215 cells. Scale Bar, 100 μm. (B) CCK8 experimental analysis of the proliferative activity of wild-type and ABHD16A KO HepG2.215 cells. (C) Representative images of wild-type and IFITM1 KO HepG2.215 cells. Scale Bar, 100 μm. (D) CCK8 experimental analysis of the proliferative activity of wild-type and IFITM1 KO HepG2.215 cells. Data are means±SEM from three independent experiments. ns, no significant difference (unpaired two-tailed *t-*test).
